# Role of Toll-Like Receptors in Neuroimmune Diseases: Therapeutic Targets and Problems

**DOI:** 10.3389/fimmu.2021.777606

**Published:** 2021-11-01

**Authors:** Haixia Li, Shan Liu, Jinming Han, Shengxian Li, Xiaoyan Gao, Meng Wang, Jie Zhu, Tao Jin

**Affiliations:** ^1^ Department of Neurology and Neuroscience Center, The First Hospital of Jilin University, Changchun, China; ^2^ Department of Neurology, Xuanwu Hospital, Capital Medical University, Beijing, China; ^3^ Department of Clinical Neuroscience, Karolinska Institutet, Solna, Sweden; ^4^ Department of Urology, The First Hospital of Jilin University, Changchun, China; ^5^ Department of Neurobiology, Care Sciences and Society, Karolinska Institute, Karolinska University Hospital, Solna, Sweden

**Keywords:** toll-like receptors, neuroimmune diseases, inhibitors, multiple sclerosis, neuromyelitis optica spectrum disorder, Guillain-Barré syndrome, Myasthenia gravis

## Abstract

Toll-like receptors (TLRs) are a class of proteins playing a key role in innate and adaptive immune responses. TLRs are involved in the development and progression of neuroimmune diseases *via* initiating inflammatory responses. Thus, targeting TLRs signaling pathway may be considered as a potential therapy for neuroimmune diseases. However, the role of TLRs is elusive and complex in neuroimmune diseases. In addition to the inadequate immune response of TLRs inhibitors in the experiments, the recent studies also demonstrated that partial activation of TLRs is conducive to the production of anti-inflammatory factors and nervous system repair. Exploring the mechanism of TLRs in neuroimmune diseases and combining with developing the emerging drug may conquer neuroimmune diseases in the future. Herein, we provide an overview of the role of TLRs in several neuroimmune diseases, including multiple sclerosis, neuromyelitis optica spectrum disorder, Guillain-Barré syndrome and myasthenia gravis. Emerging difficulties and potential solutions in clinical application of TLRs inhibitors will also be discussed.

## Introduction

Toll-like receptors (TLRs), as type 1 transmembrane protein receptors, recognize pathogen-associated molecular patterns (PAMPs) and damage-associated molecular patterns (DAMPs) and then initiate immune responses (1, 2). TLRs can be activated following the recognition of lipopolysaccharides (LPS), lipoproteins, flagellin, viral and bacterial nucleic acids, leading to a combination of protein complexes and activating chromatin remodeling and transcription factors (3). Due to a variety of regulatory functions, TLRs are actively involved in the secretion of inflammatory mediators, cellular proliferation and survival (3, 4). TLRs can also bind to extracellular domains and undergo conformational changes following dimerization to recruit intercellular downstream signaling adaptors. As each TLR paralogue perform distinct functions, various heterodimers may play different roles (3). The activated TLRs triggers a cascade of cytokine and chemokine productions, contributing to the initiation and progression of cancer (5), rheumatic diseases (6), atherosclerosis (7), neurodegenerative disease (8) and autoimmune disease (9). TLRs are necessary for protecting against diseases by accelerating the healing process to restore immune homeostasis. However, excessive TLRs activity might lead to chronic and unrestricted inflammatory responses, which could aggravate diseases.

Neuroimmune diseases can be divided into the central and peripheral nervous disorders, such as multiple sclerosis (MS), neuromyelitis optica spectrum disorder (NMOSD), Guillain-Barré syndrome (GBS), and others, such as myasthenia gravis (MG). These conditions pose a threat to the human health all over the world. It has been determined that dendritic cells, circulating monocytes, Natural Killer (NK) cells, microglia/macrophages, T and B lymphocytes are involved in the pathogenesis of neuroimmune diseases (10–12). However, the precise pathogenesis of neuroimmune diseases remain largely unknown. Although immunomodulatory drugs have achieved some success in clinical practice, most of them are non-selective immunosuppressive or cytotoxic. Limited clinical efficacy and significant side effects were found in some patients (13, 14).

Accumulating evidence has revealed that TLRs play vital roles in the pathogenesis of neuroimmune diseases, and relieved clinical symptoms were observed in preclinical models by regulating TLRs (15, 16). However, clinical evidence is still insufficient (17). How to specifically target cellular type-specific function of TLRs signaling pathway in neuroimmune disease remains unclear. Here, we updated knowledge about the role of TLRs in neuroimmune diseases, and proposed several approaches to overcome obstacles for the application of TLRs inhibitors in clinical treatment.

## Biological Characteristics of TLRs

### Structure and Function of TLRs

The molecular weights of TLRs range from 90 to 150 kDa (9). The structure of TLRs can be divided into three parts: extracellular region, transmembrane region and intracellular region. Canonically, TLRs are an important pattern-recognition receptor (PRR), which consist of extracellular leucine-rich repeats (LRRs) to recognize PAMPs and DAMPs (18, 19). PAMPs are highly conserved structural components derived from microorganisms that consist of LPS, peptidoglycan, flagellin, lipoproteins and microbial nucleic acids (20, 21). Most DAMPs are endogenous molecules released from dying cells upon cellular stress or tissue damage (22, 23). The transmembrane TLRs are well-known PRRs, which function through extracellular ligand recognition. Due to its homology with the signaling domains of interleukin (IL)-1R family members, the intracellular domain structure of TLRs is known as the N-terminal cytoplasmic Toll/IL-1 receptor (TIR) mediating homotypic interactions and facilitating downstream signaling (18, 24, 25).

To date, 13 active members of the TLRs family have been identified in mammals, including 10 in humans (TLR1-10) and 12 in mice (TLR1-9 and TLR11-13) (26). TLRs are largely categorized into two subfamilies based on their localization, such as transmembrane and intracellular regions (21). Specifically, TLR1, TLR2, TLR5, TLR6 and TLR10 are located on the cell surface, while TLR3, TLR7, TLR8 and TLR9 are mainly located on the intracellular endosome membrane. The locations of TLR4, TLR11, TLR12 and TLR13 are controversial, with being expressed on the cell membrane or intracellular endosome membrane ​according to different cell types. They may also be expressed in both cell membrane or intracellular endosome membrane (18, 21, 27, 28). The extracellular domain of each TLR corresponds to a specific PAMP, and the collective TLR family can specially recognize different pathogens. For example, TLR4 recognizes LPS and TLR10 can sense influenza A virus infection (29).

### TLR Signaling Pathway

**Figure 1:**
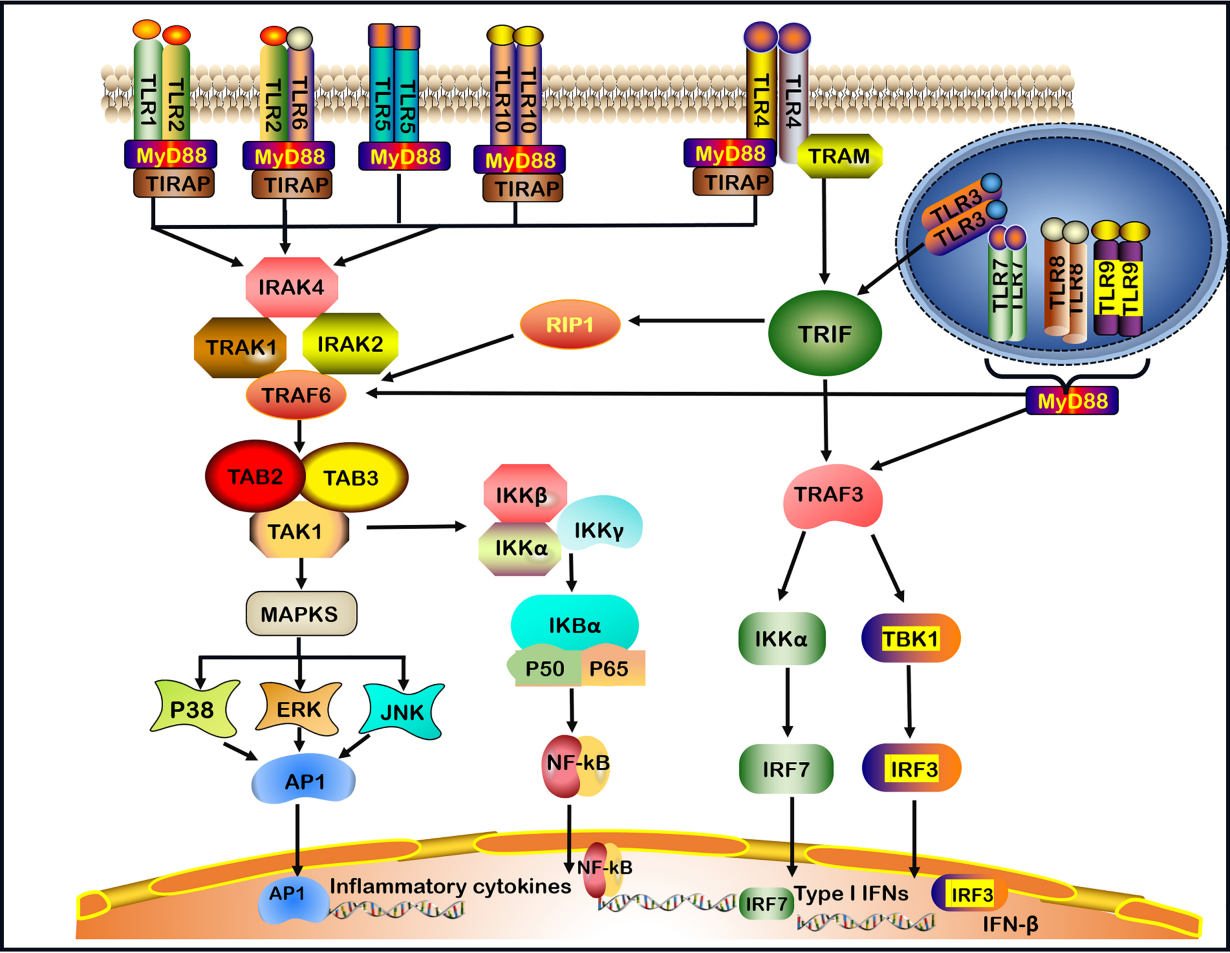
TLRs signaling pathways. TLRs (Toll-like receptors) recognize invading microbes and activate signaling pathways, which regulate immune and inflammatory responses. TLR1, TLR2, TLR5, TLR6, and TLR10 are located on the cell surface.TLR3, TLR7, TLR8, and TLR9 are located on the intracellular endosome membranes. All TLRs, with the exception of TLR3, by the (MyD88)- dependent signaling pathway. In addition, TLR4 signaling takes place in both MyD88-dependent and the MyD88-independent signaling pathway. In MyD88-dependent signaling pathway, the leucine-rich repeats (LRR) region of TLR binds to ligands resulting in the formation of TLRs heterodimer, such as TLR2-TLR1/TLR2-TLR6/TLR7-TLR8 heterodimer or TLR4/TLR9 homodimer, which induces the recruitment of the TIR domain-containing adaptor protein (TIRAP)/MyD88/interleukin-1 receptor-associated kinase-1 (IRAK-1)/IRAK2/IRAK-4 complex. After that, the complex continues to activate tumor necrosis factor receptor-associated factor 6 (TRAF6) and subsequent transforming growth factor-beta-activated kinase 1 (TAK1), TAK1-Binding Protein-1(TAB1) and TAK1-Binding Protein-2(TAB2), leading to the activation of mitogen-activated protein kinases (MAPKs, including subsequent activation of P38, ERK, and JNK) and nuclear factor-kappa B (NF-κB) signaling pathway, and promoting the production of pro-inflammatory cytokines. In the MyD88-independent signaling pathway, the activation of TLR3 or TLR4 can recruit TRIF. In particular, TLR4 requires a TRIF-related adaptor molecule (TRAM) for the activation of TRIF. Then, TRIF activates receptor-interacting protein 1(RIP1) and interacts with TRAF6 to promote subsequent inflammation signaling pathways. In addition, TRIF activates TRAF3, which in turn induces the activation of IRF3 and IRF7 to produce IFN-β and Type I IFNs respectively. Finally, TLR7, TLR8, TLR9 activate TRAF3 or TRAF6 and subsequent signaling pathways through the MyD88-dependent pathway.

TLRs recruit five cytosolic TIR domain-containing adaptors, including MyD88, TIR domain-containing adaptor-inducing IFN-β (TRIF), TIR domain-containing adaptor protein (TIRAP, also known as MAL), TRIF-related adaptor molecule (TRAM) and sterile α- and armadillo-motif-containing protein (SARM) (30, 31). TLRs signaling molecules induce the expression of proinflammatory cytokines *via* two main pathways: MyD88-dependent signaling pathway and MyD88-independent TRIF pathway (32). TLRs, with the exception of TLR3, utilize the MyD88-dependent signaling pathway. TLR4 is the only TLR that divides into MyD88-dependent and MyD88-independent (TRIF-dependent) signaling pathways (1, 2) (**Figure 1**).

When the LRR region of TLR bounds a ligand in the MyD88-dependent pathway, the TIR of the TLR receptor becomes allosteric and recruits MyD88 by interacting with the TIR domain-containing adaptor protein (TIRAP), also known as the MyD88 adaptor-like (Mal) protein (33). The binding of the C-terminal region of MyD88 results in a structural deformation of the N-terminal region of MyD88, which in turn activates the IL-1 receptor-associated kinase-4 (IRAK-4) participating in the recruitment, phosphorylation and degradation of IL-1 receptor-associated kinase-1 (IRAK-1) and IRAK-2. Then, IRAKs and MyD88 combine the tumor necrosis factor receptor-associated factor 6 (TRAF6) (1, 34, 35). TRAF6 is needed to generate the Lys63-linked ubiquitin (K63-Ub) chains, which activates the complex comprising TGF-β activated kinase 1 (TAK1), TAK1‐binding protein 1 (TAB1) and TAB2 (1, 9, 35). One pathway leads to the activation of the activator protein 1 (AP-1) through activating mitogen-activated protein kinase (MAPK). MAPK comprises three subfamilies: extracellular signal-regulated kinase (ERK) 1 and 2, c-Jun N-terminal kinase (JNK) 1 and 2, as well as p38 (36). The second pathway leads to the activation of the inhibitor of kappa B kinase (IKK) complex (IKK-α, IKK-β, and IKK-γ), causing the phosphorylation of the inhibitor of the nuclear factor κappa B (IκB) protein (1) (**Figure 1**). Finally, the nuclear factor κappa B (NF-κB) is translocated into the nucleus, where it initiates the transcription of inflammatory cytokines and molecules, such as IL-1β, IL-6, IL-8, IL-12, IL-17, tumor necrosis factor (TNF)-α, interferon (IFN)-γ, inducible nitric oxide synthase (iNOS) and intercellular adhesion molecule-1 (ICAM-1) (9, 37).

In the MyD88-independent pathway, the TIR domain of TLR3 can directly interact with TRIF. However, TLR4 requires TRAM for the activation of TRIF (38). Subsequently, receptor-interacting protein 1 (RIP1) and TRAF6 interact with TRIF to activate NF‐κB to induce proinflammatory cytokines production. In addition, TRAF family members-associated NF-κB activator (TANK) binding kinase 1 (TBK1) alongside TRAF3 phosphorylates interferon regulatory factor-3 (IRF-3) and interferon regulatory factor-7 (IRF-7) to induce the expression of IFN-β and Type I IFNs (1, 38). TLR7, TLR8 and TLR9 activate TRAF3 or TRAF6 and the subsequent signaling pathways through the MyD88-dependent pathway, induce the production of Type I IFNs and inflammatory cytokines (39) (**Figure 1**).

## Role of TLRs in Neuroimmune Diseases

Neuroimmune diseases are characterized by inflammation associated with neuron or axonal damage, loss of myelin sheath, and damage of neuromuscular junctions. Inappropriate or excessive activation of TLR signals may lead to neuroimmune diseases. Recent findings on the role of TLR in MS, NMOSD, GBS and MG will be discussed.

### Role of TLRs in MS

MS is a progressive autoimmune disease of the central nervous system (CNS), characterized by various clinical manifestations including motor and sensory deficits, visual disturbances and autonomic dysfunction (40). Experimental autoimmune encephalomyelitis (EAE) is a widely used animal model of MS. Pathological hallmarks of MS/EAE are composed of monocytes, CD4^+^ and CD8^+^ T cells, and B cells surrounding the venules and mediating myelin disintegration, axon loss and neuronal damage (41, 42).

**Figure 2:**
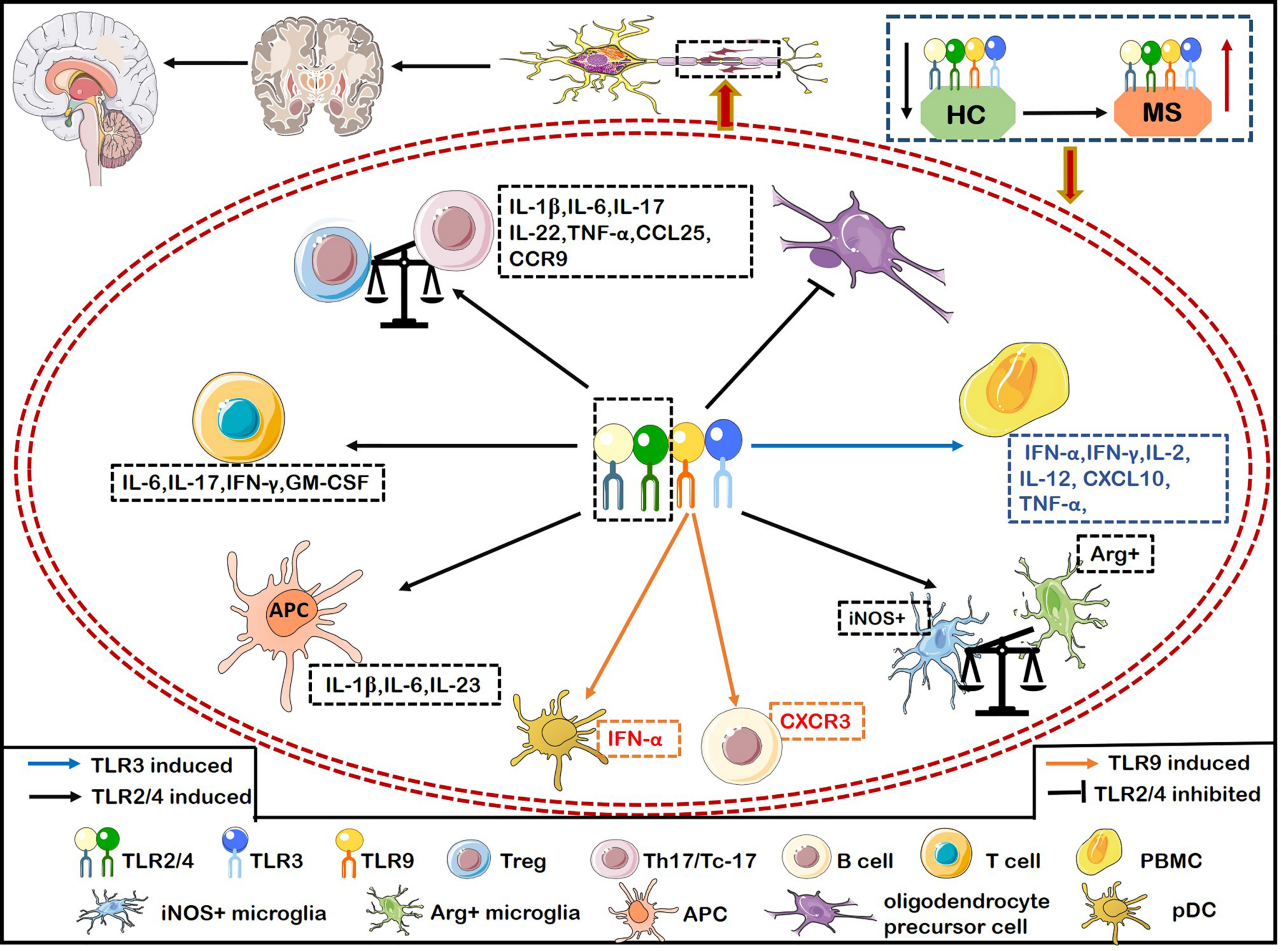
The inflammatory role of TLRs causes demyelination and hinders myelin regeneration in MS. Activation of TLR2/4 on T cells from MS induces the secretion of IFN-γ and IL-6, and activation of TLR2 alone also induces the secretion of IL-6, IFN-γ, IL-17, and granulocyte-macrophage colony-stimulating factor (GM-CSF). Activation of TLR2/4 on APCs induces more productions of IL-1β, IL-6, and IL-23. The expression of TLR2 on Treg breaks the balance of Treg/Th17 to exacerbate MS by transforming into a pro-inflammatory Th17-like phenotype. TLR2 is also up-regulated in oligodendrocytes of MS patients, which inhibits the maturation of oligodendrocyte precursor cell (OPC), resulting in the failure of remyelination in MS. The up-regulated expression of TLR2 on microglia increases the iNOS^+^ phenotype and decreases the Arg^+^ phenotype. The increased expression of TLR2/4 on Th17/Tc-17 cells promotes the up-regulation of IL-1β, IL-6, IL-17, IL-22, and TNF-α. Meanwhile, TLR4 expression on Th17 promotes the secretion of IL-17, IFN-r, CCL25, and CCR9, thereby aggravating inflammation and infiltration into CNS. Activation of TLR3 on PBMCs upregulates the production of IFN-α, IFNγ, IL-2, IL-12, TNF-α and CXCL10 to aggravate MS. Activation of TLR9 on B cells significantly increases the expression of T-bet to enhance the pathogenicity of B cells, and up-regulates the expression of CXCR3, thus promoting the inflammatory response of peripheral blood B cells and infiltrating into the CNS in MS.

TLRs play a crucial role in the pathogenesis of MS. Malika and colleagues (43) demonstrated that the expression of TLR3 and TLR4 was significantly increased in the areas surrounding inflammatory vessels and the center of MS lesions (43). TLR2 and TLR4 are actively related to the pathogenesis of MS. For example, the expression of TLR2, TLR4, and TLR9 on CD4^+^ and CD8^+^ T cells was significantly higher in patients with relapsing remitting MS (RRMS) than healthy individuals (44). The proportion of TLR^+^ (including TLR2, TLR4, and TLR9) Th17 cells and Tc-17 cells producing IFN-γ or IL-6 were positively correlated with the number of active brain lesions and neurological dysfunction by evaluating with expanded disability status scale (EDSS) and the number of active brain lesions by magnetic resonance imaging (MRI) scan (44) (**Figure 2**). Interestingly, the ligand of TLR2 (Pam3Csk4) induces more proinflammatory cytokines including IL-6, IFN-γ, IL-17 and GM-CSF than the ligand of TLR4 (LPS) and TLR9 [oligodeoxynucleotide (ODN)] from CD4^+^ and CD8^+^ T cells of MS (**Figure 2**). CD4^+^ T cells activated by Pam3Csk4 are more closely related to MS disease activity (44). Stimulation of TLR2 agonist promoted the differentiation, proliferation of Th17 cells *in vitro*, while inhibiting the expression of TLR2 on CD4^+^ T cells dramatically relieved the symptoms of EAE (45). The ligands lipopeptides and LPS bind to the TLR2 and TLR4 respectively, which adversely affect MS by increasing the production of IL-1β, IL-6 and IL-23 in antigen presenting cells (APCs) (46) (**Figure 2**). The expression of TLR2 in Treg cells from MS patients is significantly increased, shifting the Treg/Th17 balance towards a proinflammatory state and then promoting the progression of MS (47) (**Figure 2**). Furthermore, TLR2 expression is also upregulated in oligodendrocytes of MS, which inhibits the maturation of oligodendrocyte precursor cell (OPC) and remyelination through the activation of the TLR2-MyD88 signaling pathway (48) (**Figure 2**). Interestingly, systemic TLR2 tolerance induced by injecting low-dose Pam2CSK4 significantly enhanced remyelination in a preclinical model of MS, which resulted in the transformation of microglia from pro-inflammatory iNOS^+^ phenotype to non-inflammatory/pro-repair Arg1^+^ phenotype (49) (**Figure 2**). TLR2 in peripheral blood mononuclear cells (PBMCs) and CD14^+^ monocytes had a strong responsiveness to Pam2CSK4 stimulation in MS, suggesting that a high activity of TLR2 in MS may contribute to the pathogenesis of MS (50). The increased frequency of Th17-like cells expressing TLRs are involved in the pathogenesis of RRMS (46). The expressions of TLR2 and TLR4 on Th17 (IL-17^+^CD4^+^ T cells)/Tc-17 (IL-17^+^CD8^+^ T cells) were significantly upregulated in MS with major depressive disorder (MDD) compared with MS (46). Importantly, the selective serotonin reuptake inhibitors (SSRIs) effectively suppressed the expression and immune responsiveness of TLR2 and TLR4 on Th17/Tc-17-like cells (46). In summary, TLR2 plays a crucial role in promoting MS and inhibiting TLR2 can exert therapeutic effects on the animal model of MS.

TLR4 expression was significantly increased in bone marrow mesenchymal stem cells (MSCs) and peripheral monocytes of MS, activating its downstream molecules STAT-1, NF-κB, P38, JNK and CREB, increasing the production of CXCL10 and promoting inflammatory responses (51). Pertussis toxin (PTX), an adjuvant in inducing EAE, depended on TLR4 signaling molecules to facilitate T cell infiltration into the CNS (52, 53). Stimulating TLR4 on DCs drives the pathogenic function of T cells and assists the establishment of EAE model (54). Mice having TLR4^-/-^CD4^+^ T cells showed inadequate EAE induction, mild clinical symptoms and few demyelinating lesions (55). The effects may be attributed to inhibiting immune effects of Th17 and Th1 cells and reducing the secretion of IL-17 and IFN-γ (55). Furthermore, the inhibition of TLR4 also downregulated the expression of CCL25/CCR9 on Th17 cells, then reducing the migration and infiltration of Th17 cells into the CNS (56). A recent study showed that simultaneous activation of TLR3 with TLR2 or TLR4 in microglia caused severe neural network dysfunction by disrupting reactive oxygen and nitrogen species (oxidant-producing enzymes, inducible NO synthase and NADPH oxidase) (57). Accumulating data showed that a significantly higher abundance of both pDCs and conventional DCs (cDCs) was detected in MS. More importantly, the altered pDCs and (cDCs) are paramount to pro-inflammatory T cell response (58). Kristof et al. found that MS-derived pDCs and cDCs stimulated by TLR4 ligand, LPS, combined with IFN-γ significantly upregulated the secretion of IL-12p70 *in vitro*, which is important for the commitment to polarize Th1 cells (59, 60). However, no difference in TLR7 ligand, IQ-induced IFN-α secretion from pDCs and cDCs were observed between MS and healthy controls (60). In addition, the expression of CD86 on cDCs was significantly up-regulated after TLRs stimulation in MS, while no difference existed in CD80 and CD86 expression of pDCs, indicating that cDCs were in a more activated state in MS (60). The amount of investigations into the role of TLRs expressed in cDCs has escalated in recent years (61, 62), however, studies focusing on the role of TLRs expressed in cDCs in MS/EAE are sparse. Both animal and clinical studies are warranted in the future to explore the detailed mechanism which could provide an insight to a better therapeutic approach.

The role of TLR4 is not limited to promote the occurrence and development of MS. Jamie and colleagues found that TLR4 activation enhanced phagocytic activity of macrophages (promoting the clearance of myelin debris) following TLR4 agonist E6020 stimulation both *in vivo* and *in vitro* (63). TLR4 blocking inhibited microglial ability to phagocytose axon debris, which was harmful for axon outgrowth (64). Yoichiro et al. confirmed that there is a synergistical co-stimulation between TLR4 and CD40 on B cells, which increases the secretion of IL-10 during the relapse phase of MS (65). The cause of these paradoxical effects of TLR4 remains unclear. We propose that it may be related to different cellular sources of TLR4, different immune microenvironments in different stages of MS, or the cross-talk between different costimulatory molecules [CD40 and DC-specific intercellular adhesion molecule-3-grabbing nonintegrin (66)] and TLR4.

TLR3, TLR7, TLR8 and TLR9 also play an irreplaceable role in the pathogenesis of MS. Elie et al. detected TLR3 polymorphism in NK cells from MS patients, and they found that the rs3775291 allele was significantly different between MS and healthy controls (HC), and the rs3775291 (C/T or T/T) increased the incidence of MS by 71% compared with the homozygous genotype (C/C). They proposed that TLR3 mutations in NK cells are associated with MS susceptibility (67), whether the same conditions can be observed in humans in other regions or countries remains to be studied. Polyinosinic-polycytidylic acid [Poly(I:C)], a TLR3 agonist, can suppress demyelination in EAE by inducing endogenous IFN-β and peripheral CC chemokine CCL2 production (68). TLR3/MyD88 independent pathway promotes the secretion of IL-27 to suppress the mature of Th17 cells (69). The lack of Cathepsin H damages TLR3-mediated IRF3 activation, inhibits IFN-β secretion from DCs and promotes Th1 cell differentiation (70). Activation of TLR3 in astrocytes induces the expression of neuroprotective mediators, including anti-inflammatory cytokines IL-9, IL-10, and IL-11, and down-regulates the secretion of pro-inflammatory cytokines IL-12 and IL-23, and inhibits gliosis and promotes neuronal survival, angiogenesis, and myelin regeneration (71, 72).

Most previous studies suggested that TLR3 mediated the neuroprotective response during inflammatory process. However, a recent study reported that TLR3 activated by polyinosinic-polycytidylic acid on proinflammatory cytokine-pretreated astrocytes significantly promoted the production of fibronectin aggregation and led to remyelination failure in MS (73). One potential mechanism is that cytokine-induced an increase in relative mRNA of EIIIA^pos^-Fn over EIIIB^pos^-Fn and a Poly(I:C)-mediated decreased in integrin affinity, which destroyed fibronectin fibrillogenesis on the cell surface (73). A similar role of TLR3 in PBMCs to promote the development of MS was recorded (74). TLR3 of PBMCs from secondary progressive MS (PMS) and benign MS (BMS) was stimulated by Poly(I:C). The mRNA expression levels of IFN-α, IFN-γ, IL-2, IL-12, TNF-α and CXCL10 in PBMCs from PMS were significantly increased (74) (**Figure 2**). Remarkably, the activation of TLR3 on PBMCs from BMS patients increased the expression of TNF-α and CXCL10, while the activated TLR3 also significantly upregulated the expression of the specific chimera SARM-1 (a negative regulator of TLR3-mediated immune response, inhibits TRIF, NF-κB, and IRF) that down-regulating pro-inflammatory cytokines (74). These results indicated that TLR3 in PBMCs plays different or even opposite roles in different MS disease stages. Although most of the early studies supported TLR3 activation exerting neuroprotective effects in the pathogenic courses of MS/EAE, we can see that TLR3 also appears to promote the pathogenesis of MS, which may be related to the activation of different cell types, ligands, different disease stages and different signaling pathways. Still, the priming conditions of activation need to be explored.

Functional defects of TLR7 on pDCs may inhibit the secretion of IFN-α by pDCs in MS, disrupting proper control of pDCs in the T-cell mediated autoimmunity (75). The expression of TLR7 mRNA in PBMCs and monocytes of MS was damaged (TLR9 signal damage was not observed), which inhibited the secretion of IL-6 and B cell activating factor (BAFF) (76, 77) that regulates the survival, differentiation and class switching of B cells. It is critical for the maintenance of peripheral B cell pool and the initiation of B-cell responses (78). However, these effects could be rescued following IFN-β treatment (79). From this perspective, IFN-β therapy and TLR7 re-activation are not conducive to suppress the immune response of MS. Yet, it has also been speculated that this mechanism may reduce virus-triggered relapses in MS (80). The activation of TLR7 on B cells in MS has an immunosuppressive effect (81). The inadequate expression of *IFNAR1/2* and *TLR7* genes in B cells are involved in decreased endogenous IFN-β secretion in RRMS. IFN-β therapy in combination with TLR7 or TLR9 agonist (Loxorubin/CpG) induced a high endogenous IFN-β expression in B cells and increased IL-10, TGF-β and IL-27 secretion in RRMS (81). TLR7-driven B cells have an abnormal immune function in MS, however, thymosin-α1 (Tα1) improved this dysregulation by reducing the production of IL-6, IL-8 and IL-1β and increasing IL-10 and IL-35 secretion (82). Furthermore, Tα1 promoted the differentiation of regulatory B cells, thereby dampening autoimmune inflammation in MS (82). In summary, TLR7 plays a double-sided immunoregulatory role in MS depending on the specific cell type.

It has been reported that damaged TLR8 signaling pathway in MS could impair the production of IL-12, suggesting that TLR8 deficiency in MS may contribute to autoimmunity (83). TLR8 deficiency leading to autoimmunity was also noted in mice (84). TLR8 participates in axonal injury by increasing the infiltration of neutrophils and leukocytes in EAE, with the signaling pathway being remained active even after focal inflammatory infiltration disappeared (85). 1,25-Dihydroxyvitamin D3 (1,25(OH)2D3) plays an anti-inflammatory role in EAE by inhibiting TLR8 in monocytes and downstream cascade signals such as MyD88, IRF-4, IRF-7 and NF-κB (86).

Dominguez et al. (87) suggested that the gene polymorphism of TLR9 (rs352162 and rs187084) is be involved in different clinical stages of MS. TLR9 on pDCs was predominantly expressed in the leptomeninges and demyelinating lesions of MS patients and enhanced the secretion of type I IFNs and IFN-α to exacerbate MS (88) (**Figure 2**). The pDCs activated with TLR9 agonists promoted both Th1 and Th17 responses and further induced EAE (89). IFN-β therapy inhibited the expression of TLR9 on pDCs, which reduced the secretion of IL-6, TNF-α and IFN-α and decreasing the pDCs activated by pathogens (88). Besides, TLR9 activation increased the expression of TLR-1, -2, -4, -5 and -8 in PBMCs from MS (90). This reflects the cross-regulation among members of the TLR family. TLR9^−/−^ mice have an incremented IL-6 production by splenocytes (91) and then decreased EAE severity (92). In fact, APCs have the ability to maintain immune tolerance when facing foreign antigen stimulation, avoiding potential autoimmune reaction (93). The activation of TLR9 on APCs can break the immune tolerance state of APCs and promotes the differentiation of T lymphocytes to Th1 cells, thus inducing the occurrence of EAE (89, 94). The expression of T-bet was significantly increased when B cells were stimulated by IFN-γ and TLR9 agonist in MS, which increased the pathogenicity of B cells. The expression of CXCR3 receptor was also obviously upregulated, which promoted the infiltration of peripheral blood B cells across the BBB to the CNS (95) (**Figure 2**).

It seems that TLR9 plays an immune-boosting role in MS/EAE. However, recent studies displayed its immunosuppressive role in MS/EAE. Decreased expression of TLR9 on memory B cells derived from MS significantly declined the production of TLR9-mediated IL-10 by B cells (96). Activation of TLR9 up-regulates the expression of downstream molecules MyD88, TRAF6 and IRF8 promoting the development and expansion of Breg/B10 cells and the secretion of IL-10 (97). Peripheral circulation CD45 cells were recruited into the CNS when TLR9 agonist CpG oligonucleotide being intrathecal injected into EAE mice, which produced a large amount of IFN-β with immunomodulatory effect to alleviate EAE (98). Equivalently, intrathecal injection of MIS416 (a TLR9 and NOD2 bispecific innate ligand) into EAE mice up-regulated type I and II IFN, IL-10, Arg-1, CCL-2 and CXCL-10, increased the proportion of myeloid and NK cells, and reduced inflammatory T cells, which alleviated the demyelination of EAE (99).

We summarized the “pro-inflammatory” role of TLRs on various immune cells of MS, which was presented in **Figure 2**. Collectively, the dual role of TLRs in MS has been gradually explored. Firstly, the cellular type of expressing TLRs is a critical factor and it would be meaningful to focus on more accurate cell subtypes to explore their roles in the future. Secondly, it may be related to disease stages and activation of different signaling pathways caused by diverse TLRs ligands. Thirdly, the cross-talk between different co-stimulatory molecules on the same cell or different cells may also determine the immunomodulatory role of TLRs. It should be admitted that the contradictory effects of TLRs are the key to hinder the application of TLRs in clinic, therefore, it is necessary to clarify the causes and conditions of the contradictory mechanism.

### Role of TLRs in NMOSD

NMOSD is an antibody -mediated autoimmune diseases in the CNS characterized by inflammation, demyelination and axonal damage (100, 101). Because of the discovery of aquaporin-4 immunoglobulin G antibodies (AQP4-IgG), clinical and laboratory-based investigations have indicated that B cells are one of the fundamental roles in NMOSD immunopathology (102, 103). Th17 cells and their related cytokines, such as IL-17 and IL-21 are also involved in the development of NMOSD by accelerating the breakdown of the BBB, facilitating inflammatory cells infiltration into CNS lesions, and collaborating with B cells to release AQP4-IgG (104). Like other neuroimmune diseases, the pathogenesis of NMOSD is associated with a number of environmental and hereditary susceptibility. Growing evidence indicated that viral and bacterial infections were associated with NMOSD (105, 106). These pathogens that caused NMOSD were recognized by TLRs and triggered the secretion of proinflammatory factors. TLRs are typically expressed in human CD4^+^ T cells. Barrosa et al. (107) found that the expression of TLR2, TLR4 and TLR9 on non-activated CD4^+^ T cells of NMOSD patients was significantly increased compared with the healthy individuals. Remarkably, the co-expression of IL-17 and IL-6 were significantly enhanced in the high expression of TLR2, TLR4, and TLR9 of Th17 cells from NMOSD, and it was positively correlated with the EDSS score. The high expression of TLRs in Th17 cells aggravated the neurological dysfunction of NMOSD through the increased secretion of pro-inflammatory cytokines (107). Furthermore, IL-17 expression was significantly increased in the high expression of TLR2 and TLR9 from activated CD4^+^ T cells, thus promoting inflammatory responses in NMOSD (107).

Intriguingly, the high expression of IL-10 in TLR2^+^ Treg cells (non-classical IL-10^+^IL-17^+^Treg cells) was found only in patients with mild neurological dysfunction of NMOSD, while a significantly lower proportion was found in patients with severe neurological dysfunction (107). This supports that function of Treg cells in NMOSD is damaged (insufficient IL-10 secretion), thus leading to sustained neuroinflammation (107–110). Interestingly, the later study found that agonists of TLR9 induced IL-10 secretion from NMOSD-derived Treg cells, while TLR2 agonist did not (111). Paradoxically, TLR2 knockout animal model of psoriasis significantly weakened the mRNA expression of foxp-3 and IL-10, inhibited the proliferation of Treg cells and exacerbated psoriasiform skin inflammation. Correspondingly, the use of TLR2 agonists promoted the production of Treg cells and the secretion of IL-10 (112). Brittney et al. also reported that the nucleic acids released by bacteriolysis triggered IL-10 secretion mostly dependent on TLR2 activation (113). The contradictory role of TLR2 in Treg cells may be related to different diseases, however, there is still lack of studies on the role of TLRs in NMOSD.

Dias et al. (111) evaluated the direct effects of different TLR ligands on CD4^+^ T cells form NMOSD and healthy individuals, their results suggested that the agonists of TLR2 (Pam3C), TLR4 (lipopolysaccharide) (LPS) and TLR5 (FLA), but not TLR9 (ODN), elevated CD4^+^ T cells expansion in NMOSD patients. As expected, Pam3C, LPS, FLA and ODN did not show the obvious CD4^+^ T cells proliferative activities in the healthy individuals (111). Besides, they found that all TLRs agonists induced the release of IL-6, IL-17 and IL-21 by CD4^+^ T cells without extra stimuli, and with TLR2 and TLR4 agonists being the most effective. Of note, this still only occurred at NMOSD-derived CD4^+^ T cells (111). This indicates that the TLRs pathway on CD4^+^ T cells has altered in NMOSD patients, at least in terms of its activity. Additionally, the agonists of TLR2 and TLR4 increased the production of Tfh cells and promoted the secretion of IL-21, which was positively correlated with neurological dysfunction of NMOSD (111).

AQP4-IgG-mediated complement-dependent astrocyte injury is recognized as the core of the pathogenesis of NMOSD (114). Kazuya et al. found that damaged astrocytes caused by AQP4-IgG can release large amounts of mitochondrial DNA (mtDNA), which promotes the release of IL-1β from mononuclear cells through the activation of TLR9 and NLRP3 inflammasome-dependent manner. Subsequently, it leads to activating leukocytes, destroying BBB and promoting the migration of monocytes into the CNS (115). A later study showed that released mtDNA from damaged astrocytes by AQP4-IgG can further induce the generation of CCL2 from astrocytes (116). mtDNA acts as a molecular bridge of innate immunity and then activates monocytes by activating TLR9, and CCL2 induces monocytes to migrate into the CNS (116).

Studies on the role of TLRs in NMOSD are not as extensive as those in MS. The roles of TLRs in B cells, DCs, and microglia/macrophages of NMOSD patients still remain unknown. TLRs are actively involved in the pathogenesis of NMOSD, and further research is required to clarify cellular and molecular mechanisms and it may shed light on novel therapeutic approaches for NMOSD.

### Role of TLRs in GBS

GBS is an immune-mediated demyelinating disorder of the peripheral nervous system (PNS). Clinical symptoms are characterized by tingling, limb weakness, autonomic dysfunction and numbness (117). Experimental autoimmune neuritis (EAN) is an animal model of GBS. The pathogenesis of GBS/EAN involve a variety of immune cells (such as T cells, B cells and macrophages) and a complex network of cytokines (118).

The TLR4 gene polymorphism (Asp299Gly) is closely related to an increased susceptibility to GBS (119). Apart from Asp299Gly, the Thr399Ile polymorphism is also associated with the incidence of acute motor axonal neuropathy (AMAN), a subtype of GBS (119). Anti-TLR4 antibodies interdicted the processes of demyelination of the PNS by inhibiting monocyte chemoattractant protein-1 production from Schwann cells (120). Du et al. detected mRNA levels of TLR2, TLR4, MyD88 and NF κB in PBMCs from patients with GBS, and found that they were significantly higher than healthy controls. Moreover, PBMCs from GBS produced more TNF−α and IL−1β after stimulation with TLR2 and TLR4 agonists (PGN and LPS), indicating that TLR2 and TLR4 expression on PBMCs is involved in the pathogenesis of GBS (121). In addition to TLR2 and TLR4 in GBS, TLR9 expression was also increased in PBMCs, which promoted the secretion of IFN-γ and positively correlated with the degree of disability of GBS (122). Paradoxically, Gries et al. evaluated TLR9 mRNA in CD4^+^ T cells and found no difference between GBS patients and healthy controls. The discrepancy may be caused by different time points of collecting PBMCs and in inconsistent disease stages of GBS. Furthermore, TLR9 may be highly expressed in PBMCs except for CD4^+^ T cells (123). It was noted that *tlr9* mRNA was upregulated in the spleen, sciatic nerve, PBMCs and lymph nodes throughout the course of EAN, suggesting that it may be involved in the pathogenesis of EAN in different disease phages (124). TLR9 can also promote the expression of IL-12 to induce the differentiation of Th1 cells, playing a role in the pathogenesis of GBS/EAN (89).

The expression of TLR2, TLR6 and TLR11 on CD4^+^ T cells, and TLR2, TLR4, and TLR6 on the major histocompatibility complex class II positive (MHCII^+^) APCs were significantly upregulated in the acute phase of GBS and EAN, while the expression of TLR1 was decreased and the secretion of IL-17A was enhanced (123). Significant upregulation of TLR2 was also observed in sciatic nerves of EAN rats, which correlated with the disease severity (125). Peripheral TLR2 signaling pathway promotes the upregulation of IFN-γ, IL-6, and IL-17 secretion, which work coordinately to increase peripheral nerve inflammation and damage the myelin sheath and axons (118, 126). The TLRs signaling can also induce the activation of self-reactive T or B cells and activate APCs through the MyD88-dependent or -independent pathways to trigger the adaptive immunity (127). Darabi et al. (128) demonstrated that APCs activated through the TLRs signal pathway, especially TLR4 and TLR9, can induce T cells differentiate into Th1 cells and result in tissue destruction. Th1 is not sufficient to induce autoimmune pathology without induction by TLR9-activated APCs. Cross-reactivity between immune cells triggered by microbial infection is critical for autoimmune response. TLRs connects microbes and immune cells, especially under the cytokine storm, microorganisms activates APCs by activating TLRs to activate the third signal, further cross-reacting with T cells to induce and activate autoimmune T cells, triggering an autoimmune response (128).

GBS is triggered by a variety of infectious or noninfectious agents (129). The Gram-negative *Campylobacter jejuni* (*C. jejuni*) is now recognized as the primary trigger of GBS. Most infections are acquired from eating raw or undercooked poultry, unpasteurized milk and contaminated water (130). It produces a variety of glycoconjugates, including human ganglioside analogs and multiple activators of TLRs, and targets MyD88, TRIF, macrophage galactose-type lectin (MGL), etc., which induces autoimmune diseases (131). Molecular mimicry between sialylated lipooligosaccharide (LOS) structures of *C. jejuni* and ganglioside epitopes on the human nerves that generate cross-reactive immune response results in an autoimmune attack on the myelin or axon of peripheral nerves in GBS (130). *C. jejuni* is resistant to proteinase digestion, inducing the activation of neutrophils and macrophages and activating NF-κB through TLR2 and TLR4 (132, 133). The initiation of GBS by *C. jejuni* was strongly dependent on its pathogenic LOS structure, which triggers the innate immune system through TLR4 signaling (133). Zeb et al. developed a vaccine against *C. jejuni* infection by genome-wide screening. The vaccine interacts with TLR4 to trigger the release of primary and secondary immune factors to enforce humoral immune response against *C. jejuni*, thereby preventing GBS (134).

Zika virus (ZIKV) has emerged as a public health threat due to its teratogenic nature and associated with the occurrence of GBS (135, 136). The mechanism of ZIKV infection causing GBS is unclear. TLRs, autophagy, apoptosis and unfolded protein response (UPR) pathways are considered as a potential mechanism (137). TLR3 could be activated by ZIKV by sensing the replication intermediate of viral RNA and was upregulated in human organoids and mouse neurospheres after ZIKV infection (138). Activated TLR3 triggered the production of proinflammatory cytokines during ZIKV infection, which upregulated the STAT3 pathway and reduced the STAT1 phosphorylation in a suppressor of cytokine signaling (SOCS)-3 dependent manner, thereby inhibiting interferon response triggered by RIG-I-like receptors (RLR) and reducing the antiviral effect (139). However, the antiviral cytokine response was enhanced following the inhibition of TLR3, while the production of proinflammatory cytokines was decreased. The cross-talk between the antiviral (RLR) and inflammatory (TLR) responses may further induces GBS (139). In addition, the TLR7/8 agonist R848 blocked the ZIKV replication in monocytes (140). Whether other TLRs can be activated by ZIKV have not yet been established. We speculate that ZIKV may be involved in the pathogenesis of GBS through TLRs. In addition, there is no vaccine against ZIKV so far. In the future, the relationship between ZIKV and TLR3 may be conducive to the development of an effective anti-ZIKV vaccine to prevent GBS.

Macrophage migration inhibitory factor (MIF) is critically involved in the pathogenesis of GBS/EAN (141). It promotes the recruitment of macrophages to the PNS and the expression of proinflammatory cytokines, including TNF-α, IL-6, IL-8, and IL-12 to damage myelin and axonal (142). However, the role of MIF begins with upregulating the expression and activation of TLR4 then promotes the translocation of NF-κBp65 into the nucleus through the MyD88-dependent/independent pathway (142, 143). Altogether, these results indicate that TLRs signaling contribute to the pathogenesis of GBS.

### Role of TLRs in the Pathogenesis of MG

MG is an acquired autoimmune disease characterized by neuromuscular junction transmission dysfunction, with main manifestations being fluctuating skeletal muscle fatigue, post-activity worsening symptoms associated with a reduction of acetylcholine receptor (AChR) clustering (144) and thymic hyperplasia featured with ectopic germinal center (145). Similar to other neuroimmune diseases, MG is associated with both humoral and cellular immunity (146). The exact mechanisms remain obscure and may be related to genetic and environmental factors. Evidence with chronic inflammation, TLRs activation, and persistent viral infection in MG is accumulating (147).

Wang et al. (148) detected the mRNA expression of whole TLRs in the PBMCs of both MG patients and healthy controls. They observed that all TLRs expression, except for TLR7, in MG patients were significantly different from those in the healthy controls. The levels of TLR1, TLR6 and TLR10 were considerably lower, whereas TLR2, TLR3, TLR4, TLR5, TLR8 and TLR9 mRNA were significantly upregulated in PBMCs from MG as compared to healthy controls. It is worth mentioning that the expression level of TLR9 mRNA has an evident positive relation with the clinical severity of MG. Thymic stromal cells such as thymic epithelial cells (TECs) and myoid cells, express all TLRs (149). TLR3 expression was higher in TECs cultures derived from the thymus of MG patients than healthy controls (16). Additionally, poly(I:C), the well-known agonist of TLR3, triggers the overexpression of α-AChR in TECs by releasing of IFN-β and anti-AChR antibodies (149). TLR4 overexpression and activation in MG TECs altered effector T cells (Teff)/regulatory T cells (Treg) balance, induced the production of Th17-related cytokines and drove DCs recruitment *via* overexpression of CCL17 and CCL22 to regulate immune cell trafficking in inflamed organs (150). TLR7 and TLR9 in MG thymus are capable of leading to abnormal B cells/plasma cells proliferation, maturation, and survival, as well as the provision of additional costimulatory signals. B cells escape from regulatory cell tolerance checkpoints depending on MyD88-depending way in MG patients once combined with the ligand like Epstein–Barr virus (EBV), which assists in the production of type I IFN and long-term inflammation (151, 152).

MiR-146a is a key modulator of innate immunity that orchestrates inflammatory signaling (153). As an inhibitor of the TLR pathway, miR-146a targets to inhibit TRAF6, IRAK1 and NF-κB to prevent inflammatory stimulation mediated by TLR overactivation (154). In addition, miR-146a deficiency promotes the activation of c-REL, which accelerates B cells proliferation, differentiation and germinal center (GC) development (155, 156). Nevertheless, miR-146a expression is significantly downregulated in hyperplastic thymus of MG and the levels of IRAK1, TRAF6 and c-REL were upregulated and negatively correlated with the level of miR-146a. Interestingly, miR-146a in the thymus of MG patients was upregulated after corticosteroid therapy, and insufficient expression of miR-146a in MG may lead to sustained TLR activation, impaired inflammatory resolution ability and thymic hyperplasia (156).

Experimental autoimmune myasthenia gravis (EAMG) is a classical experimental model for MG, which is induced by a purified antigen of AChR solubilized in complete Freund’s adjuvant (CFA) containing heat-inactivated mycobacterium tuberculosis (MTB). LPS, the TLR4 activator, has been confirmed to be efficient to replace MTB (16). TLR3’s agonist Poly(I:C) can induce transient phenomenon that express α-AChR, IFN-β and chemokines such as CXCL13 and CCL21, leading to the B-cell recruitment in EAMG (157). Although the EAMG is associated with muscle weakness due to anti-AChR antibody attack, it does not fully reproduce MG disease, because the thymus does not present ectopic GC development (158) [a classic pathological feature of MG: abnormal thymus with features of tertiary lymphoid organs, includes new angiogenesis processes, overexpression of inflammatory cytokines and chemokines, and invasion of B cells leading to ectopic GC development (159, 160)]. Fortunately, this was significantly reversed when TLR3 combined with TLR4 agonist was used in EAMG induction, however, the agonists of TLR7 and TLR9 do not have this effect. The agonist of TLR3 combined with TLR4 not only formed GC, but also prolonged symptom duration and increased CXCL13 secretion (157). Another contradictory research showed that inhibition of the TLR9 pathway by the oligodeoxynucleotide (ODN) H154 in EAMG decreased Tfh cells and B cells of GC, abated anti-AchR antibody production and terminally alleviated clinical symptoms (161). We propose that the agonists and inhibitors of TLR9 may not maintain completely opposite results. Although TLR9 does not contribute to the formation of ectopic GC, it acts on the inside of the Tfh and the B cells after the formation of GC. Overall findings indicated that inappropriate TLRs signaling activation may participate in the pathogenesis of MG/EAMG and more experimental studies are needed to confirm it.

## Targeting TLRs Signaling Pathway for the Treatment of Neuroimmune Diseases

### TLRs and Existing Drugs

The current treatments of neuroimmune diseases mainly include plasma exchange (PE), intravenous immunoglobulin (IVIg), application of glucocorticoid and immunosuppressive agents. Natalizumab prevents the infiltration of leukocytes into the CNS by inhibiting α4 integrin, thereby alleviating the autoimmune response of MS (162). However, the strong immunosuppression declined the ability of the immune system to monitor, potentially increasing the risk of progressive multifocal encephalopathy (PML) (163). Surprisingly, TLR3 agonists could re-establish CNS immune surveillance in EAE when α4 integrin was inhibited and reduced the risk of PML, suggesting that natalizumab therapy in combination with TLR3 agonists may restore a more appropriate immune balance in MS (163). Fingolimod (FTY720) is the first modulator of sphingosine 1-phosphate receptors (S1PR) to receive regulatory approval for relapsing-remitting MS. S1P1 is critical for the regulation of lymphocyte transport. However, Fingolimod reduces the outflow of lymphocytes from lymph nodes mediated by S1P, resulting in lymphocyte circulation depletion (164). TLR4 activation enhances the expression of chemokines through the transactivation of S1P-S1PR. FTY720 inhibited the synthesis and released proinflammatory chemokine CXCL5, CXCL10, and CCL2 that induced by TLR4 activation on astrocytes and microglia (165). Mycophenolate mofetil (MMF) is an inhibitor of inosine-5’-monophosphate dehydrogenase, which is a prodrug of mycophenolic acid (MPA). MMF inhibits the proliferation of T and B lymphocytes by depleting their guanosine nucleotides, weakening the immune response and antibody formation (166). A recent study has shown that MPA can dose-dependently downregulate the expression of CD80 and CD86 (markers of DC maturation) activated by TLR7 and TLR9 on mDCs and reduce IL-12 secretion in systemic lupus erythematosus (SLE). In pDCs, MPA inhibited IRF7 nuclear translocation and IFN-α secretion by strongly inhibiting AKT activity (167).

Despite above evidence, there is still a poor prognosis or serious sequelae after therapy for neuroimmune disorders. Exploring new therapeutic methods is still an urgent need. The receptors, adapter molecules and key kinases in TLRs signaling pathway can be considered as therapeutic targets. Development of TLRs antagonists is a promising direction in therapy neuroimmune diseases, which has been summarized in **Table 1**.

**Table 1:** Inhibitors of TLRs signaling pathway.

Inhibitors	Targets	Object	Functions	References
Phloretin	TLR2/1	Human embryonic kidney (HEK) 293-hTLR2 cells	Suppresses TNF-α and IL-8 production	(164)
OPN-305	TLR2/1, TLR2/6	CD14(+)CD45(+) cells (monocytes)	Decreases IL-6 production	(165)
C16H15NO4 (C29)	TLR2/1, TLR2/6	HEK-hTLR2 cells and THP-1 macrophage-like cell line	Decreases IL-1β and IL-8 production	(166)
AP177	TLR2	HEK293 cells, HEK293T, TLR2-HA, THP1-Blue cells,	Decreases IL-6 and IL-8 production	(167)
NI-0101	TLR4	Phase I study evaluated NI-0101 in healthy volunteers	A dose-dependent inhibition of IL-6, TNF-α, CXCL10, IFN-β	(168)
TAK-242	TLR4/TIRAP/TRAM	HEK293 cells, RAW264.7 cells	Decreases TNF-α, IL-6, and NO production	(169)
CPG-52364	TLR7, TLR8, TLR9	Clinical trials of RA	Inhibits disease development in RA	(170)
IMO-8503	TLR7, TLR8, TLR9	lung and pancreatic cancer cells	Treatment of cancer cachexia	(171)
IMO−8400	TLR7, TLR8, TLR9	Phase 2 trial in moderate-to-severe plaque psoriasis	Reduces psoriasis severity	(172)
TAC5 series	TLR3, TLR7, TLR8,TLR9	RAW 264.7 cells, mouse model of psoriasis and SLE	Inhibits the secretion of IL-6, IL-17, TNF-α	(173)
PF-06650833, BAY1834845, BAY1830839, CA-4948	IRAK4	Clinical trials of RA and SLE (RA dominated)	Inhibits the secretion of IL-1, IL-6, TNF-α	(174–176)
HS-243	IRAK-1, 4	Human rheumatoid arthritis fibroblast-like synoviocytes.	Inhibits the expression of IL-8, CCL5, CXCL12	(177)
ST2825	MyD88	mouse model of nonreperfused acute myocardial infarction (AMI).	Decreases IL-6 production	(6)
RDP58	MyD88,	EAE	Reduces cellular infiltration within the spinal cord and TNF-α expression levels	(178)
MG132, BAY117082	NF-κB	Leukemia cells	Arrests the process of leukemia cells differentiation cycle and induces apoptosis in leukemia cells	(179)
ML120B, PS-1145	IKK-2	Human pulmonary cells and primary human bronchial epithelial (HBE) cells	Decreases the expression of intercellular adhesion molecule (ICAM)-1 and IL-8	(180)
PHA-408	IKK-2	Rat model of arthritis	Inhibits TNF-α production	(181)

### Emerging Drugs

#### TLR2 Inhibitors

Phloretin is an inhibitor of TLR2/1 heterodimerization and can suppress the secretion of TNF-α by blocking the TLR2 signaling pathways. It is a naturally occurring dietary flavonoid that is abundant in fruits (168). OPN-305 is the first humanized IgG4 monoclonal antibody against TLR2 by targeting the ligand-binding site, preventing heterodimerization of the receptor with TLR1 or TLR6 and decreasing the production of IL-6 (169). Pragnesh et al. (182) found that the compound C16H15NO4 and a derivative ortho-vanillin inhibited the TLR2/1 and TLR2/6 signaling pathway induced by synthetic and bacterial TLR2 agonists in human HEK-TLR2 and THP-1 cells (182). AP177, an antagonist of TLR2, manifests a therapeutic potential in disordered TLR2 immune responses conditions by significantly inhibiting NF-κB activity and decreasing the secretion of cytokines IL-6 and IL-8 (183).

#### TLR4 Inhibitors

TLR4 is a promising therapeutic target for the treatment of neuroimmune diseases. NI 0101, an anti-TLR4 antibody, can potentially block any TLR4 ligands without obvious safety concerns. The phase I study of healthy volunteers confirmed that it inhibited the production of IL-6, TNF-α, CXCL10 and IFN-β in a dose-dependent manner with well-tolerated (170). TAK-242, a small molecule specific inhibitor of TLR4, disrupts the interactions of TLR4 with its adaptor molecules TIRAP and TRAM (171). However, the application of TAK-242 in phase III clinical trials for the treatment of severe sepsis was terminated, because it did not significantly decrease the cytokine levels in patients with severe sepsis and septic shock (69). Of note, Chaperonin 10 (Cpn10) was applied in a phase II trial in patients with MS. The expression levels of proinflammatory cytokines and new gadolinium-enhancing lesions were not significantly different between two groups with 5 mg or 10 mg weekly, respectively (172, 173).

#### TLR7, TLR8, and TLR9 Inhibitors

CPG-52364, a derivative of chemical compound quinazoline with a small molecular weight, inhibits the activation of TLR7, TLR8, and TLR9 and is well tolerated in clinical trials of psoriasis and rheumatism (RA) (174). IMO-8503, another TLR7, 8, and 9 antagonist, can act as a potential treatment for cancer cachexia (175). IMO−8400, an oligonucleotide-based antagonist of TLRs 7, 8, and 9, can reduce psoriasis severity with severe adverse events (176). IMO−3100 (inhibits TLR7 and TLR9) and IRS 661 (inhibits TLR7) had been considered to be potential therapeutic compounds (18, 69). A new TAC5 series of compounds with small molecule (TAC5 and its derivatives TAC5-A, TAC5-C, TAC5-D and TAC5-E) were possible candidates for the treatment of autoimmune diseases, which effectively inhibited TLR3, TLR7, TLR8 and TLR9 signaling pathways, and significantly also inhibited the activation of NF-κB, decreased the phosphorylation of MAPK and declined the secretion of TNF-α and IL-6 (184). These findings indicate their enormous potential to treat neuroimmune diseases.

#### Other Inhibitors of TLRs Signaling Pathway

**Figure 3:**
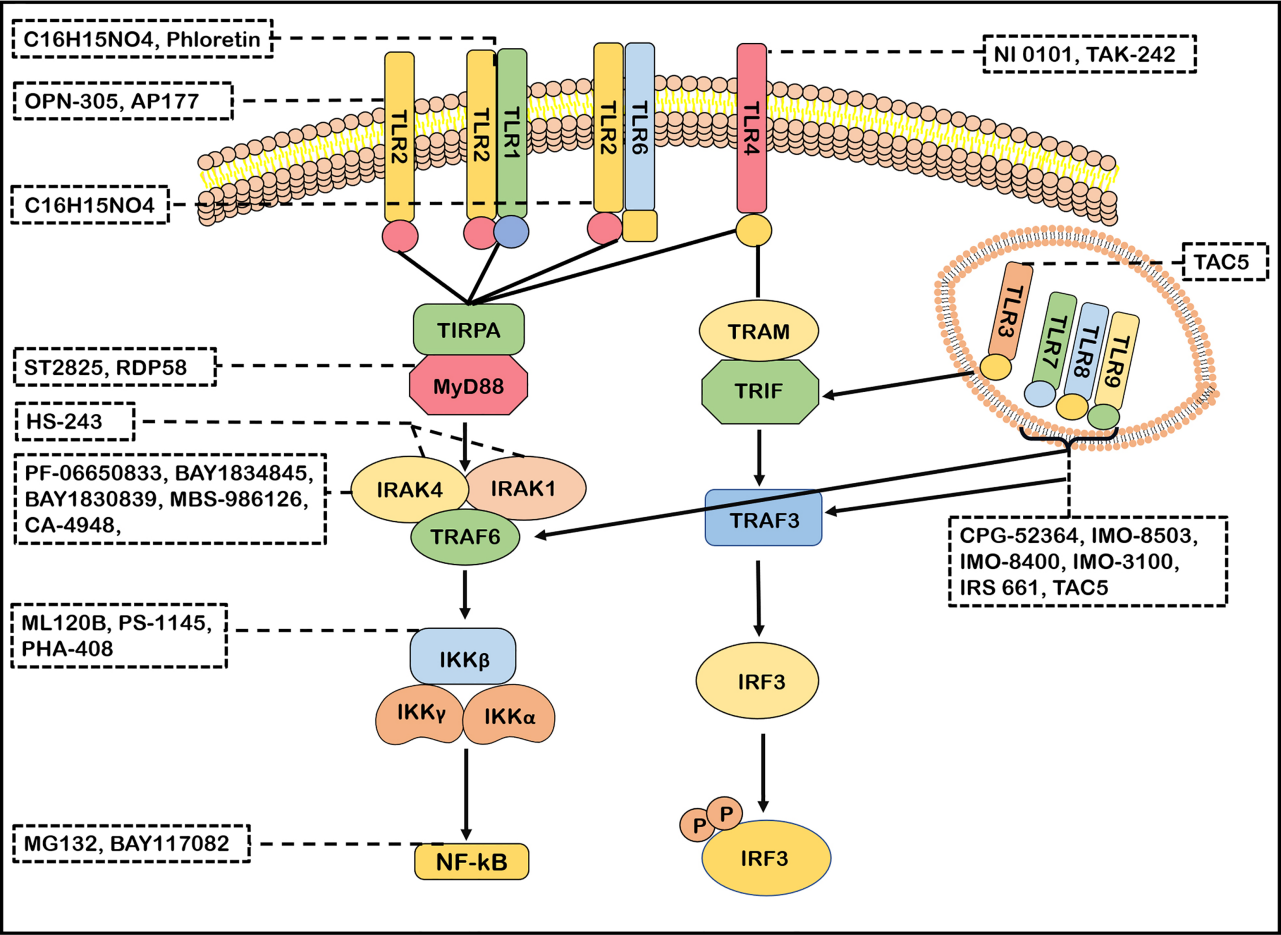
Antagonists of TLRs signaling pathways. Many antagonists have been developed against TLRs. Phloretin is an inhibitor of TLR2/1, OPN-305 and AP177 block TLR2 signaling. C16H15NO4 inhibits the TLR2/1 and TLR2/6 signaling pathway. TAK-242 and NI 0101 disrupt the interactions of TLR4 with its adaptor molecules TIR domain-containing adaptor protein(TIRAP). IMO-8503, CPG-52364, IMO-8503, IMO−8400, IMO−3100 and IRS 661 are the antagonists of TLR7, 8, 9. TAC5 effectively inhibits TLR3, TLR7, TLR8 and TLR9 signaling pathways to prevent the production of inflammatory factors. ST2825 and RDP58 inhibit myeloid differentiation primary response 88(MyD88)dimerization, interfering with recruitment downstream molecules. PF-06650833, BAY1834845, BAY1830839, CA-4948 and MBS-986126 are antagonists of IRAK4 and effectively block its follow-up effect. HS-243 exquisite selectively inhibits IRAK-1/4. ML120B, PS-1145 and PHA-408 are the inhibitors of IKK-β. MG132 and BAY117082 inhibit nuclear factor-kappa B (NF-κB) so that inflammatory response cannot be activated.

TLRs signaling pathways has been identified as a potential therapeutic target for neuroimmune diseases. The effects of molecular inhibitors and antibodies ameliorating the malfunction of innate immune caused by abnormal TLRs signaling pathway were explored (21). Inhibitors of IRAK4, such as PF-06650833, BAY1834845, BAY1830839, CA-4948 and MBS-986126, have been tested in autoimmune diseases (6, 177). Importantly, PF-06650833, BAY1834845, BAY1830839 and CA-4948 are now used in clinical trials of RA and SLE with therapeutic effects and a favorable safety (6, 177, 178). MBS-986126 reduced inflammation in preclinical models of autoimmune diseases, achieving remarkable results (179). HS-243, a highly effective IRAK inhibitor, selectively inhibits IRAK-1/4 (185). These data support a continued evaluation in clinical trials of TLRs inhibitors for the treatment of autoimmune diseases. ST2825 could inhibit MyD88 dimerization, interfering with the recruitment of IRAK1 and IRAK4 (180). RDP58 treatment decreased cellular infiltration in the spinal cord of EAE (181). The proteasome inhibitor peptide MG132 has been attributed to inhibiting NF-κB through the inhibition of IκB degradation. BAY117082 is an NF-κB inhibitor *via* inhibiting the IκB phosphorylation (186, 187). ML120B, PS-1145 and PHA-408 are the inhibitors of IKK-β which is a protein subunit of IκB kinase (188, 189). Taken together, many inhibitors targeting TLRs signaling pathways have been developed (**Figure 3**). However, few blockers have been used in clinical application for treatment in neuroimmune diseases. However, the blockers with minor side effects and high efficiency need to be developed.

### Unsolved Problems

There are still certain problems in the application of TLRs inhibitors for clinical treatments. First, the contradictory role of TLRs in immune cells becomes a significant obstacle. As mentioned above, the conflicting role of TLR3, TLR4, TLR7, TLR8 and TLR9 in different immune cells implied that total suppression of a certain TLR signal molecules may not only inhibit immune regulatory effects of TLR, but also damage the regeneration and repair of axons, myelin sheaths and even neurons. In addition, the cross-talk between the same TLR on different cells or different TLRs on the same cell fails to precisely suppress excessive immune responses TLRs caused. Second, for the same TLR on a cell, different TLR ligands may lead to the activation of different signaling pathways, resulting in conflicting effects (71). For example, Theiler’s murine encephalomyelitis virus (TMEV) infection activated TLR3 signaling pathway, which promoted the production of CCL2, CXCL8 and proinflammatory cytokines through the activation of NF-κB and IRF-3 (190). However, the activated TLR3 by poly I:C did not show the same results *in vitro*. This was attributed to the cytoplasmic dsRNA-activated kinase PKR by TMEV infection, but this cannot be achieved by poly I:C stimulation. These results highlight the difference between the TLR3 response induced by direct nonlytic virus infection and by extracellular poly I:C stimulation (191). There are unknown molecules that regulate different signaling pathways after the activation of the same TLR in a complex microenvironment *in vivo*. The sweeping inhibition of TLR is not only one-sided but may bring harmful side effects. Therefore, finding new regulatory targets on the TLR pathway with a targeted drug delivery is crucial for the application of TLRs into clinical practice.

## Future Manipulation for The Treatment of Neuroimmune Disorders

### Regulation of Gut Microbiome

Epigenetic modification is an important mechanism leading to gene expression changes. The relationship between the microbiota-gut-brain axis and CNS diseases is a classic example. Human microbiota and its metabolites adjust immune cells and cytokines partial by epigenomic modifications to participate in the occurrence and development of CNS diseases (192). TLR, as PRRs expressed by a variety of cells in the gastrointestinal tract, directly binds to the microbiome to maintain intestinal homeostasis or induce gut dysbiosis resulting in inflammatory responses (193). Emerging evidences support that TLRs are involved in the pathogenesis of neurodegenerative diseases through the gut-brain axis possibly, including MS. The imbalance of the intestinal microbiome not only affects the expression level of TLRs in APCs, but also leads to the imbalance of Th17/Treg cells (192, 194). Therefore, regulating TLRs through modulating microbiota may be a safe and effective method for the treatment of neuroimmune diseases in the future.

In recent years, several studies have identified diet as one of the major factors shaping the composition of the gut microbiome, thereby affecting systemic immune systems (195). High-calorie diets and lacking physical exercise can upregulate cellular metabolism toward biosynthetic pathways and lead to the gut dysbiosis, altered intestinal immunity state and low-level systemic inflammation. Instead, low-calorie diets act on nuclear receptors and enzymes that upregulate oxidative metabolism, downregulate synthesis of proinflammatory molecules, and restore or maintain a healthy gut symbiotic microbiota (196). Modulation of the intestinal microbiota provides an opportunity for the treatment of neuroinflammatory diseases (195). Nutrients may play a role in inflammation by regulating the expression of TLRs, proinflammatory and anti-inflammatory cytokines, thus interfering with the crosstalk and signal transduction of immune cells (197). For instance, retinoid supplementation has been shown to decrease inflammatory responses by downregulating TLRs expression and secretion of proinflammatory cytokines TNF-α and IL-6 during macrophage phagocytosis (198). Vitamin B2 inhibits the activation of TLR4 and TLR6 on macrophages stimulated by LPS and zymosan, and decreased the production of TNF-α and iNOS, while increased the secretion of IL-10 (199). Vitamin D3 inhibits the expression of TLR2 and TLR4 proteins and downregulates the production of TNF-α in monocytes in a time- and dose-dependent manner (200). Moreover, regulating the microbiome by dietary amino acids affects the TLRs regulation of macrophages and DCs, and impacts the gut-microbiome-immune (201).

The intake of probiotics might improve intestinal dysbacteriosis and reduce gut leaky, further lowing the production of inflammatory mediators, decreasing the activation of macrophages and DCs and mitigating inflammatory reactions of MS (202). Another study reported that probiotics downregulated the TLRs/MyD88/NF-κB signaling pathway, promoting the secretion of M2 polarization factors (IL-10 and IL-4) and inhibiting M1 polarization factors (TNF-α, IFN-γ, IL-1β, iNOS, COX-2, and IL-6) in the type 2 diabetes (203).

TLR2 plays a crucial role in linking the microbiome to MS. Recently, Nicholas proposed the hygiene hypothesis that reduces neuroinflammation and improves myelin repair by injecting sufficient microbial-derived molecules Lipid 654 [L654: produced by bacteroidetes and present in the serum of healthy people and MS patients, but at lower levels in MS patients (204)] into the circulation of MS patients to repeatedly stimulate TLR2 for a long time (205). Although many studies have been conducted on the role of TLRs in neuroimmune diseases, satisfactory results have not been achieved. Previous experimental methods resulted in the expression of TLRs in an ‘all-or-none’ state (205). Either failure to suppress TLR, or complete suppression of TLR leads to loss of protective immune response in the face of infection. We need to find ways to regulate TLRs expression and its immune effects at an appropriate range. In future studies, we should try to find some easily regulated ‘control buttons’ between TLRs and neuroimmune diseases, such as the microbiome.

### Application of Nanotechnology

Although many TLR inhibitors have been developed, they have not been used for clinical treatments mainly due to its inadequate response (206), especially with traditional delivery methods: insufficient stability, poor water solubility, injection site aggregation, not lasting effect, systemic toxicity, and nonspecific immune cell suppression (207–209). Developing accurate targeted drugs and effective delivery method are the most important issue. With the rapid development of material chemistry research in recent years, the application of innovative biomaterials and drug delivery devices may address these problems. Nanocarriers have often been used for drug delivery, with common nanocarriers being inorganic carriers (metal nanocrystals or carbon nanomaterials) and organic carriers (polymer nanocarriers or liposomes) (210). Nanoparticles-submicron-sized drug carriers have been actively investigated for the delivery of antibiotics, nucleic acids, peptides/proteins and chemotherapeutics (211). Polymer nanoparticles can mediate passive or active targeted drug transport, improve the drug concentration of lesions, and the stability of loading drugs. By changing the size of the polymer nanoparticles, the clearance of small drug molecules from the kidney or liver can be reduced, thereby increasing the drug cycle time (210, 212). By controlling drug release ability, the systemic side effects induced by drugs can be reduced significantly (210, 212). Polymeric nanoparticles fabricated with poly (d,l-lactide-co-glycolide, PLGA) is a biocompatible polymer, which was stable in saline and small enough to be administered by subcutaneous or intramuscular injections. It had been used as an efficient delivery platform for TLR7/8 agonists, enhancing DCs uptake and facilitating lymphatic drainage (211). We believe that the use of ‘nano-TLRs inhibitors’ in neuroimmune diseases may achieve more ideal therapeutic effects, which may greatly reduce global immunosuppressive effects in the circulation, and precisely reach the lesion center, improve absorption rate and reduce adverse drug reactions. Nanocarriers can realize targeted drug delivery for a specific cell type. For example, TLRs agonists are delivered to macrophages by nanocarriers in tumors, which promoted macrophages from M2 to M1 phenotype with an anti-tumor effect (213). Furthermore, 1-1000 nm nanoparticles were preferentially phagocytosed by APCs (214). Therefore, TLRs agonists are accurately delivered to DCs through nanoparticles to promote its maturation, playing a role of stronger antigen presentation and promoting immune responses (215, 216). The combination of TLRs inhibitors and nanocarriers would be a promising approach, which may improve therapeutic effects and reduce potential side effects.

## Conclusions

TLRs inhibitors, as a potential immunotherapy, have been widely studied in a variety of autoimmune diseases (217, 218), but not in neuroimmune diseases such as NMOSD, GBS, MG and MS. Of note, the previous studies regarding TLRs in neuroimmune diseases mainly focused on cellular experiments *in vitro* and TLR expression levels in immune cells of patients. TLRs agonists have achieved promising results in tumors. Thus, we are confident that TLRs signaling pathways will also be used as therapeutic targets in neuroimmune diseases in the future, especially combining the application of next-generation sequencing and novel material chemistry technology.

## Author Contributions

HL and SL drafted the manuscript. JH, SXL, XG, and MW edited and revised the manuscript. JZ and TJ designed the framework and revised the manuscript. All authors read and approved the final manuscript.

## Funding

This work was supported by grants from the General Program of the National Natural Science Foundation of China (No. 81671177), Natural Science Foundation of Jilin Province Science and Technology Development Plan Project (20190201043JC), Key Research and Development Project of Social Development Division of Jilin Science and Technology Department (20200403109SF), Special Project for Health Professionals of Jilin Provincial Finance Department (JLSWSRCZX2020-0056), as well as the grants from the Swedish Research Council(No. 2015-03005) and grants from the First hospital, Jilin University of China.

## Conflict of Interest

The authors declare that the research was conducted in the absence of any commercial or financial relationships that could be construed as a potential conflict of interest.

## Publisher’s Note

All claims expressed in this article are solely those of the authors and do not necessarily represent those of their affiliated organizations, or those of the publisher, the editors and the reviewers. Any product that may be evaluated in this article, or claim that may be made by its manufacturer, is not guaranteed or endorsed by the publisher.
